# A purified energy-converting hydrogenase from *Thermoanaerobacter kivui* demonstrates coupled H^+^-translocation and reduction *in vitro*

**DOI:** 10.1016/j.jbc.2022.102216

**Published:** 2022-06-30

**Authors:** Alexander Katsyv, Volker Müller

**Affiliations:** Department of Molecular Microbiology & Bioenergetics, Institute of Molecular Biosciences, Johann Wolfgang Goethe University, Frankfurt am Main, Germany

**Keywords:** acetogenic metabolism, extremophile, energy-converting hydrogenase (Ech), proton translocation, proteoliposomes, *Thermoanaerobacter kivui*, [4Fe-4S], iron–sulfur cluster, ACMA, 9-amino-6-chloro-2-methoxyacridine, DCCD, N,N-dicyclohexylcarbodiimide, DDM, n-dodecyl β-D-maltoside, Ech, energy-converting hydrogenases, ETH2120, N,N,N′,N′-tetracyclohexyl-o-phenylendioxydiacetamid, Fd, ferredoxin, Fd^2-^, reduced ferredoxin, [Ni-Fe], hydrogenase active site, PFOR, pyruvate:ferredoxin oxidoreductase, TCS, 3,3′,4′,5-tetrachlorosalicylanilide, TPP, thiamine pyrophosphate

## Abstract

Energy-converting hydrogenases (Ech) are ancient, membrane-bound enzymes that use reduced ferredoxin (Fd) as an electron donor to reduce protons to molecular H_2_. Experiments with whole cells, membranes and vesicle-fractions suggest that proton reduction is coupled to proton translocation across the cytoplasmatic membrane, but this has never been demonstrated with a purified enzyme. To this end, we produced a His-tagged Ech complex in the thermophilic and anaerobic bacterium *Thermoanaerobacter kivui*. The enzyme could be purified by affinity chromatography from solubilized membranes with full retention of its eight subunits, as well as full retention of physiological activities, *i.e.*, H_2_-dependent Fd reduction and Fd^2-^-dependent H_2_ production. We found the purified enzyme contained 34.2 ± 12.2 mol of iron/mol of protein, in accordance with seven predicted [4Fe-4S]-clusters and one [Ni-Fe]-center. The pH and temperature optima were at 7 to 8 and 66 °C, respectively. Notably, we found that the enzymatic activity was inhibited by N,N′-dicyclohexylcarbodiimide, an agent known to bind ion-translocating glutamates or aspartates buried in the cytoplasmic membrane and thereby inhibiting ion transport. To demonstrate the function of the Ech complex in ion transport, we further established a procedure to incorporate the enzyme complex into liposomes in an active state. We show the enzyme did not require Na^+^ for activity and did not translocate ^22^Na^+^ into the proteoliposomal lumen. In contrast, Ech activity led to the generation of a pH gradient and membrane potential across the proteoliposomal membrane, demonstrating that the Ech complex of *T. kivui* is a H^+^-translocating, H^+^-reducing enzyme.

Energy-converting hydrogenases (Ech) are a group of membrane-bound enzyme complexes that couple oxidation of reduced ferredoxin (Fd) with the reduction of protons and vice versa (1, 2, 3, 4, 5). They are widely distributed in the anaerobic world (1, 3, 6, 7), were low-potential Fd (E^0^′ = −450 to −500 mV) are reduced by metabolic enzymes such as Fd-dependent glyceraldehyde-3-phosphate dehydrogenase (8), pyruvate:Fd oxidoreductases (9, 10), CO dehydrogenases (11, 12) or hydrogenases (13, 14, 15, 16). Oxidation of reduced Fd with reduction of protons to hydrogen gas is exergonic and data obtained with whole cells or vesicle systems are in accordance with the hypothesis that the redox energy liberated is used to pump out H^+^ of the cells (17, 18, 19, 20, 21). The established H^+^ gradient is then used to drive the synthesis of ATP (17, 18, 19). In some heterotrophic bacteria, this chemiosmotic mechanism will lead to additional ATP synthesis by the cell while the majority of ATP is synthesized by substrate-level phosphorylation (16, 22, 23). In autotrophic organisms such as some acetogenic bacteria Fd is reduced with H_2_ by an electron-bifurcating hydrogenase and the following re-oxidation of reduced Fd by Ech is the only way to synthesize net ATP (16, 17, 22). This allows for the synthesis of only a fraction of an ATP per mol of product (acetate) formed, but enough to sustain microbial life at thermodynamic equilibrium (22, 24).

The reaction catalyzed by Ech is bidirectional, and indeed, there are a couple of metabolic scenarios where Ech is the motor to drive the endergonic reduction of Fd with H_2_ as electron donor (16, 25). In soluble electron-bifurcating hydrogenases the energy for the endergonic reaction is supplied by bifurcating electrons to a second, electropositive acceptor (13, 16); in Ech complexes the driving force is assumed to be the transmembrane electrochemical ion gradient (17, 19). Ech catalyzes H_2_-dependent Fd reduction in some methanogens (26) and also in acetogens (16).

Ech complexes are composed of a minimum of six subunits and have extensively been studied in methanogenic archaea (4, 27, 28). In addition to the hydrogenase large and small subunits, energy-converting hydrogenases contain at least two additional hydrophilic subunits and two integral membrane subunits (2, 4). These six subunits form the basic structure of Ech's and are conserved in all members of this hydrogenase subfamily (1, 6). Moreover, the six-subunit core shares high sequence similarities with hyperthermophilic membrane-bound hydrogenases (Mbh, Mbs) (29, 30, 31, 32) or the complex I as found in electron transport chains from bacteria to humans (33, 34, 35, 36). The evolutionary relationship of Ech complexes to complex I has been addressed in recent reviews (33, 37, 38, 39, 40).

An interesting observation is that any species analyzed this far has two gene clusters each encoding an Ech complex (3). This raises the question of whether one or both are ion pumps or one maybe not coupled to ion transport (3, 17). Since ion transport has never been determined for a purified Ech complex, due to the instability of the enzymes and low yields after purification, we have used the previously established genetic system for the thermophilic acetogenic bacterium *Thermoanaerobacter kivui* to produce a tagged version of the Ech2 complex and to purify the complex in comparatively high yield and activity by affinity chromatography in one step. This allowed us to address the question of ion transport in Ech2 with a purified enzyme reconstituted into liposomes.

## Results

### Purification of genetically modified Ech2 from *T. kivui*

**Figure 1:**
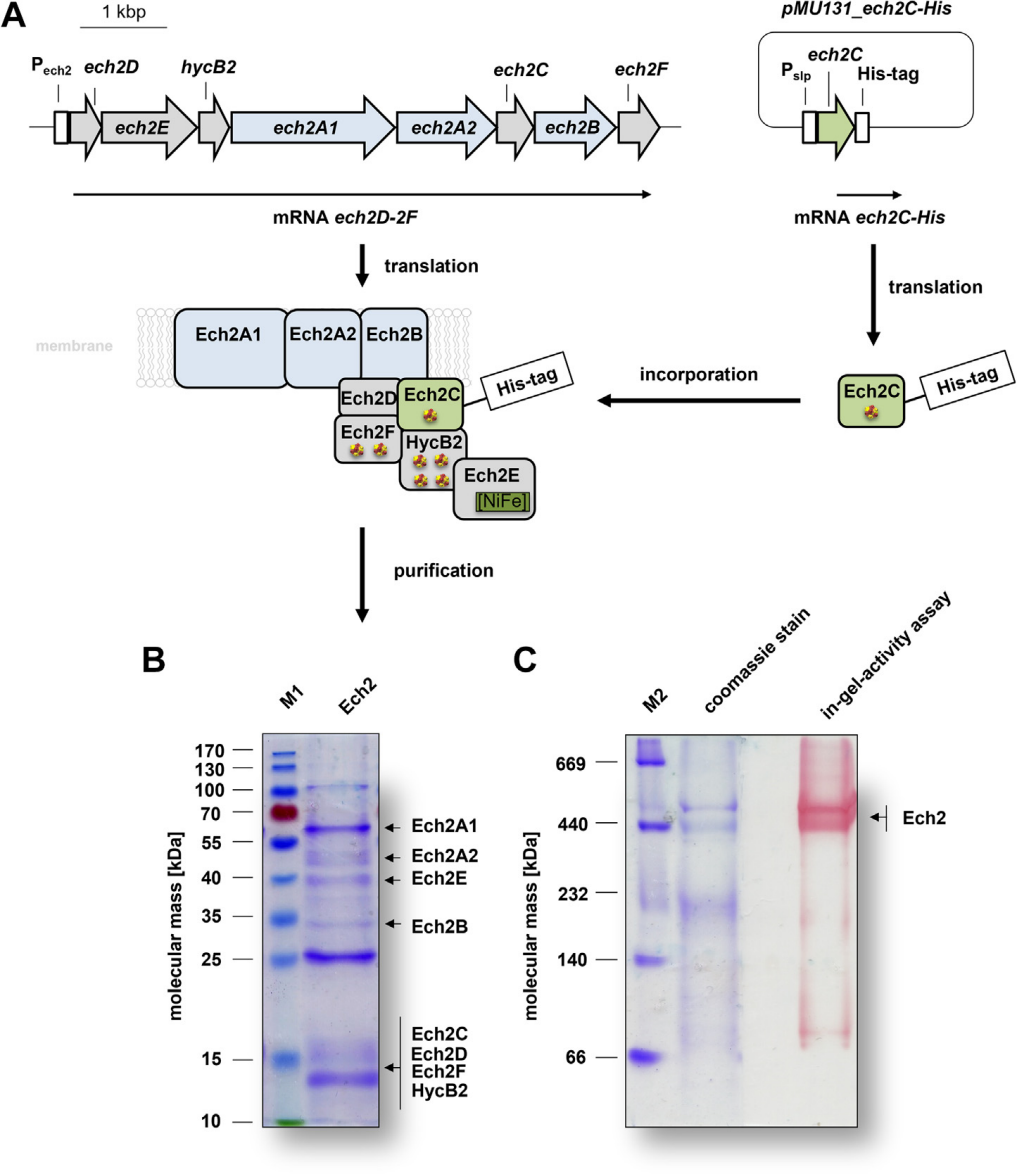
**Purification of Ech2.***A*, to purify a tagged version of Ech2 the plasmid *pMU_ech2C-His* was transformed in *T. kivui*. The expression of *ech2C-His* was under control of the constitutively expressed S-layer promoter (P_slp_). The *in trans* produced Ech2C-His assembled into the Ech2 complex encoded by the genome. The genetically modified Ech2 complex was purified *via* affinity chromatography. Purified Ech2 was separated by SDS- (*B*) or SDS-free PAGE (*C*) and stained with Coomassie *Brilliant Blue* G250. Ten micrograms of protein was applied to each lane. *C*, hydrogenase activity of Ech2 was determined with triphenyltetrazolium chloride and methylviologen under an atmosphere of 3% hydrogen. M1, prestained page ruler; M2, high-molecular-weight calibration ruler.

To produce a tagged version of Ech2 the gene encoding *ech2C* was fused with a 10× His-tag encoding sequence to the 3′ end and cloned into the expression vector *pMU131* (10, 41). The expression of *ech2C-His* was under control of the constitutively expressed S-layer promoter (P_slp_) (41). The plasmid *pMU131_ech2C-His* was transformed in *T. kivui* in the hope that the *in trans* produced His-tagged subunit Ech2C would assemble into the complex encoded by the genome and allow for the purification of the entire complex *via* affinity chromatography (Fig. 1*A*). To this end, cells were grown on glucose, membranes were prepared, proteins were solubilized with n-dodecyl β-D-maltoside (DDM) (2.5 mg DDM/mg protein) and the solubilisate was applied to a Ni^2+^-NTA column. Indeed, the entire Ech2 complex was eluted from the column (Fig. 1). Using this procedure the enzyme was purified 148-fold with a specific Fd^2-^:H^+^ oxidoreductase activity of 7.4 U/mg and with a Fd-dependent hydrogen evolution activity of 1.6 ± 0.3 U/mg. The yield was low with an average of ≈0.05 mg/g wet cells (Table 1). Analyses of the purified Ech2 separated on a 12% SDS-polyacrylamide gel revealed 10 proteins with apparent molecular masses of ≈100, 60, 48, 40, 35, 25, and 18 to 12 kDa (Fig. 1*B*). These molecular masses correspond well with the expected sizes for Ech2A1 (TKV_c19720, 69.4 kDa), Ech2A2 (TKV_c19710, 42.6 kDa), Ech2B (TKV_c19690, 35.3 kDa), Ech2C (TKV_c19700, 15.4 kDa), Ech2D (TKV_c19750, 13.9 kDa), Ech2E (TKV_c19740, 41 kDa), Ech2F (TKV_c19680, 17.5 kDa), and HycB2 (TKV_c19730, 12.5 kDa) of *T. kivui*. All subunits of Ech2 in the preparation were identified by peptide mass fingerprinting. However, peptide mass fingerprinting also revealed a high abundance of a 58-kDa chaperon (TKV_c05620), 103-kDa protein translocase secA (TKV_c05070), and a 25-kDa purine nucleoside phosphorylase (deoD; TKV_c16190) in the preparation sample, which are not expected to influence the Ech-catalyzed reaction.

**Table 1:** Purification of Ech2 from *T. kivui*

Purification step	Total volume [ml]	Protein concentration [mg ml^−1^]	Total protein [mg]	Specific activity^a^ [U mg^−1^]	Purification [-fold]	Yield [%]
membranes	40	4.3	172	0.05	1	100
Ni^2+^-NTA	1	0.32	0.32	7.4	148	0.2

Shown is a representative purification of Ech2 out of 6 g wet cells.

a Ech activity was measured with Fd as electron donor and H^+^ as electron acceptor.

The predicted mass of an Ech2 monomer with one copy for each subunit is 248 kDa. Surprisingly, the native PAGE revealed three molecular masses of ≈230, 450, and 520 kDa for the purified complex, but the in-gel activity assay showed that only the ≈450 and ≈520-kDa complex had a H_2_:triphenyltetrazolium chloride oxidoreductase activity; this is consistent with Ech2 being a dimer with slightly different subunit compositions (Fig. 1*C*). Furthermore, we identified 34.2 ± 12.2 mol of iron/mol of protein, which matches the prediction that Ech2 contains seven [4Fe-4S] and one [Ni-Fe]cluster.

### Basic biochemical properties of Ech2

**Figure 2:**
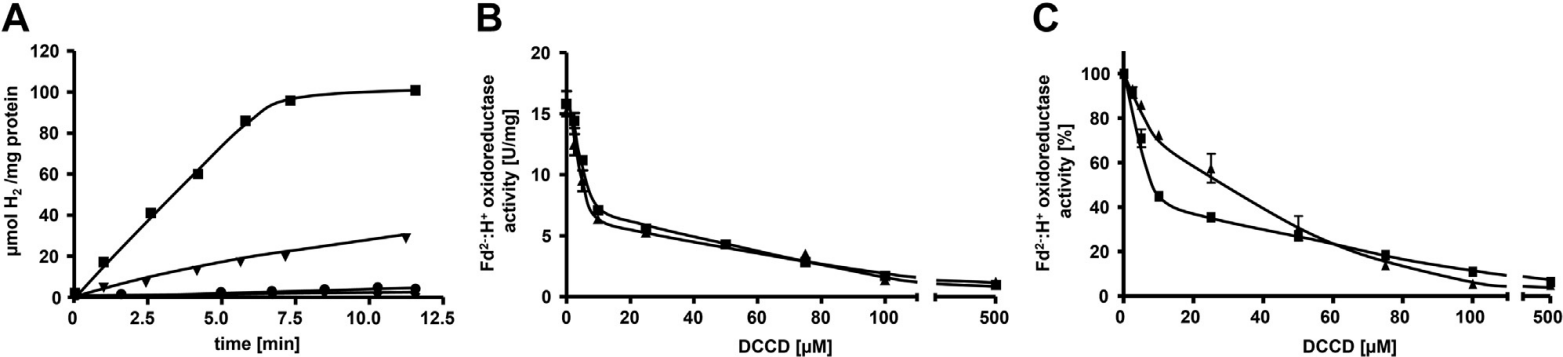
**Ech2 activity is strongly inhibited by DCCD.***A*, H_2_ production was measured in an assay mixture containing buffer E and 15 μg Ech2 in the presence of Fd (■), Fd + 100 μM DCCD (▼), or in the absence of Fd (●) or Ech2 (♦) as described in Experimental procedures. *B*, for inhibition studies 15 μg desalted Ech2 (final Na^+^ concentration in the assay: ≈180 μM) was preincubated in buffer H for 20 min at room temperature in the presence (▲) or absence (■) of 50 mM NaCl with 0 to 500 μM DCCD, respectively. *C*, inhibition of DCCD at pH 6 (▲) compared with pH 7.5 (■) was tested in buffer I or H. Fd^2-^:H^+^ oxidoreductase activity was determined as described in Experimental procedures. H_2_ was measured *via* gas chromatography as described previously (17). All data points are mean ± SEM; N = 3 independent experiments.

The purified Ech2 complex catalyzed H_2_ production from reduced Fd as electron donor in the presence of a continuous Fd reduction system with an average activity of 10.4 ± 3.4 U/mg (Fig. 2*A*) (10). When Fd or Ech2 was omitted from the assay H_2_ was not produced (Fig. 2*A*). Furthermore, Ech2 catalyzed the reverse reaction, the reduction of Fd with H_2_ as electron donor with an average activity of 1.6 ± 0.3 U/mg (Fig. S1).

Next, we assessed key biochemical properties of the purified Ech2 complex including the temperature and pH profile as well as substrate affinities. To ensure an ideal reflection of the physiological conditions, we exclusively used the Fd^2-^:H^+^ and H_2_:Fd oxidoreductase assay to determine Ech2 activity. Ech2 was active at temperatures ranging from 22 to 90 °C with a maximal Fd^2-^:H^+^ and H_2_:Fd oxidoreductase activity of 7.5 ± 0.8 U/mg and 1.6 ± 0.2 U/mg at the optimal growth temperature of *T. kivui* (66 °C) (Fig. S2, *A* and *B*). Ech2 activity was also temperature dependent and decreased by 77 or 88% at 22 °C and by 62 or 63% at 40 °C (Fig. S2, *A* and *B*). At 90 °C the activity decreased by 76 or 63% (Fig. S2, *A* and *B*). The pH range was relatively narrow with 27, 41, and 0% Fd^2-^:H^+^ oxidoreductase activity at pH 6, 9, and 10 or 15, 46, and 45% H_2_:Fd oxidoreductase activity at pH 6, 7, and 10 (Fig. S2, *C* and *D*). However, Ech2 had the highest Fd^2-^:H^+^ oxidoreductase activity of 8.5 ± 0.4 U/mg at pH 8 (Fig. S2, *C* and *D*) and H_2_:Fd oxidoreductase activity of 1.3 ± 0.1 U/mg at pH 9 (Fig. S2, *C* and *D*). At pH 5 Ech2 activity was completely abolished (Fig. S2, *C* and *D*).

Furthermore, we determined the K_m_ values for all reaction partners of Ech2. The dependence of the H_2_:Fd oxidoreductase reaction on Fd and H_2_ was hyperbolic with saturation at 30 μM Fd and 318 μM H_2_ (in the aqueous phase), respectively (Fig. S3, *A* and *B*). The K_m_ values of Ech2 for H_2_ and Fd were 45.0 ± 7.8 μM and 17.8 ± 4.3 μM, respectively (Fig. S3, *A* and *B*). The absence of H_2_ or Fd led to a complete loss of activity. Because *T. kivui* is able to convert CO as sole carbon and energy source, we analyzed whether the membrane-bound hydrogenase is inhibited by CO. Therefore, we tested Fd^2-^:H^+^ oxidoreductase activity in the presence of different CO concentrations (in the aqueous phase) (Fig. S4). Indeed, the enzyme was inhibited by CO and 50% inhibition was observed with 204.7 ± 5.3 μM CO, indicating a high tolerance for CO *in vitro*, compared with other hydrogenases (13, 16, 42).

Last, we assessed the impact of ions on the Fd^2-^:H^+^ oxidoreductase activity. So far, the nature of the chemiosmotic coupling ion for Ech2 remains enigmatic (17). When Na^+^ was omitted from the assay (contaminating Na^+^ concentration ≈180 μM), the specific activity was 10.0 ± 1.4 U/mg. However, the addition of Na^+^ or Li^+^ did not stimulate the activity at all, demonstrating that Ech2 activity does not depend on Na^+^.

### Ech2 is strongly inhibited by DCCD

Several membrane proteins involved in ion transport are inhibited by N,N-dicyclohexylcarbodiimide (DCCD), which binds covalently to carboxylates buried in the membrane phase and thereby inhibits H^+^ or Na^+^ binding and transport (43, 44, 45). DCCD also inhibited Fd^2−^:H^+^-oxidoreductase activity of Ech2. The maximum inhibition occurred at 500 μM DCCD with a residual Ech2 activity of only 18% of the control (Fig. 2, *A* and *B*). Furthermore, the inhibition by DCCD was dose dependent and 50% inhibition was observed at pH 7.5 with 9.9 ± 0.4 μM and at pH 6 with 21.8 ± 2.7 μM DCCD (Fig. 2, *B* and *C*). Interestingly, DCCD inhibition was not relieved by the addition of 50 mM Na^+^ before starting the reaction, as observed in Na^+^-translocating ATP synthases (46, 47, 48, 49, 50, 51). However, when Ech2 was preincubated with 0 to 500 μM DCCD and 50 mM Na^+^ simultaneously, DCCD inhibition was not relieved as well (Fig. 2*B*). These data indicate that DCCD and H^+^ compete for a common binding site.

### Reconstitution of Ech2 into liposomes

**Figure 3:**
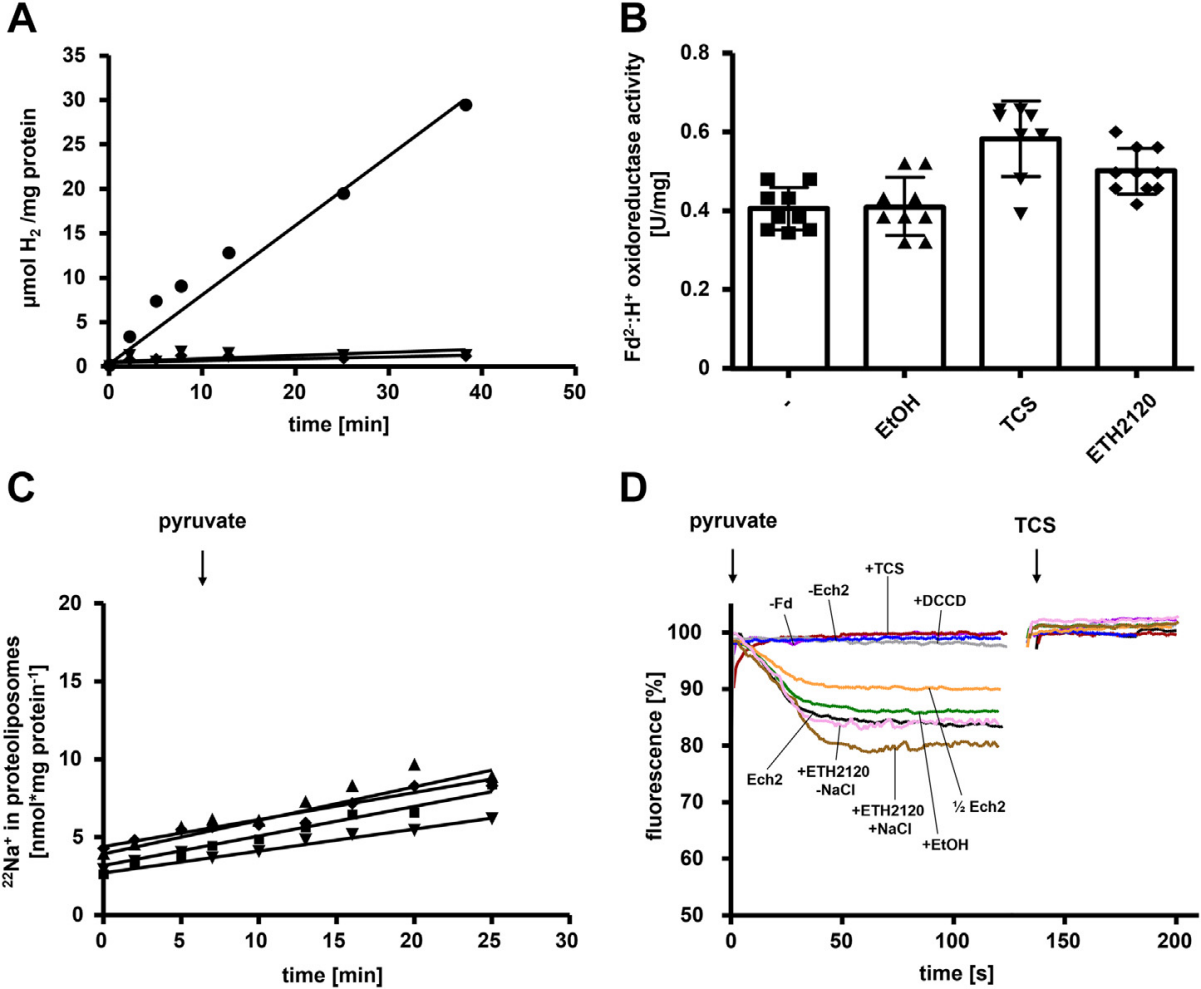
**Ech2 activity establishes a pH gradient across the membrane.***A*, H_2_ production was measured in an assay mixture containing buffer D in the presence of 240 μg proteoliposomes and Fd (●) or in the absence of Fd (▼) or proteoliposomes (♦) as described in Experimental procedures. H_2_ was measured *via* gas chromatography as described previously (17). *B*, for inhibition studies 100 to 250 μg proteoliposomes were preincubated with 30 μM TCS, ETH 2120, or 1% [v/v] ethanol for 20 min at room temperature in buffer D (including 10 mM NaCl) before the Fd^2-^:H^+^ oxidoreductase reaction was started. *C*, the generation of a ΔpNa^+^ was measured in an assay mixture containing buffer D, 950 μg proteoliposomes preincubated with 30 μM TCS (▼) or ETH 2120 (♦), in the presence (■) or absence of Fd (▲) as described in Experimental procedures. The Fd^2-^:H^+^ oxidoreductase reaction was started with the addition of 10 mM pyruvate as indicated. Radioactivity was determined as described previously (61). *D*, the generation of a ΔpH was recorded by measuring the fluorescence (excitation: 410 nm, emission: 490 nm) of the pH indicator ACMA as described in Experimental procedures. To induce the establishment of a ΔpH, the assay contained buffer D and 240 (*black*) or 120 μg (*orange*) proteoliposomes and the Fd^2-^:H^+^ oxidoreductase reaction was started with the addition of 10 mM pyruvate as indicated. To dissipate the electrical field 30 μM TCS was added as indicated. In control assays Fd was omitted (*purple*), liposomes without reconstituted Ech2 (*blue*) were used, or proteoliposomes were additionally preincubated with 1% [v/v] ethanol (*green*), 50 μM DCCD (*gray*), 30 μM ETH2120 and 10 mM NaCl (*brown*), 30 μM ETH2120 without the addition of NaCl (*pink*) or 30 μM TCS (*red*), respectively. All data points are mean ± SEM; N = 3 independent experiments.

To determine H^+^ or Na^+^ translocation coupled to H_2_ evolution with the purified enzyme, we reconstituted the purified Ech2 complex into liposomes. First, we confirmed that the H_2_-forming activity of Ech2 was still present after reconstitution into the liposomes. Therefore, the proteoliposomes were washed after the final preparation step and the Fd^2-^:H^+^ oxidoreductase activity was determined. The Ech2 proteoliposomes had an average specific activity of 0.54 ± 0.11 U/mg with reduced Fd as reductant (Fig. 3, *A* and *B*). In contrast, there was no activity when Fd or Ech2 was omitted from the assays. In sum, these data demonstrate a functional and stable incorporation of Ech2 into the liposomes (Fig. 3*A*). It is important to note that the liposomes were prepared from L-α-phosphatidyl-choline and are thus unstable at the temperature optimum of Ech2. Therefore, all measurements were performed at 40 °C, where the Fd^2-^:H^+^ oxidoreductase activity was 10% of the one under optimal conditions at 66 °C. Second, we tested whether the active site of Ech2 faced the lumen or the medium. Therefore, the vesicles were disrupted by 1-butanol and the H_2_-evolving activity was measured. Proteoliposomes without any addition of 1-butanol had a Fd^2-^:H^+^ oxidoreductase activity of 0.54 ± 0.11 U/mg compared with 0.60 ± 0.12 U/mg in the presence of 1-butanol. According to these experiments 90% of the active site protruded outward of the proteoliposomes.

To evaluate the membrane integrity of the proteoliposomes at 40 °C, an artificial H^+^ gradient was established across the lipid layer and it was analyzed whether proteoliposomes could hold such an artificial pH gradient. Therefore, proteoliposomes with reconstituted Ech2 were loaded with NH_4_Cl and subsequently diluted in a fluorescence cell containing ammonium-free choline buffer and 9-amino-6-chloro-2-methoxyacridine (ACMA) as pH indicator. The dilution led to the dissociation of NH_4_Cl into NH_3_ and H^+^, followed by diffusion of NH_3_ across the membrane, leading to an acidification within the liposomes (ΔpH) and thus a fluorescence quench of ACMA (52). The quenching was indeed observed with the proteoliposomes and the pH gradient was stable for 20 min (Fig. S5). The pH gradient could be fully dissipated upon addition of 1-butanol (Fig. S5). Thus, the procedure led to energetically intact proteoliposomes.

To test whether Ech2 activity is coupled to the generation of a Na^+^/H^+^ potential across the vesicle membrane, the proteoliposomes were incubated in the presence of the protonophore 3,3′,4′,5-tetrachlorosalicylanilide (TCS) or the Na^+^ ionophore N,N,N′,N′-tetracyclohexyl-o-phenylendioxydiacetamid (ETH2120) that disrupts electrochemical H^+^ and Na^+^ potentials, respectively. If Ech2 activity leads to the generation of an electrochemical field, enzymatic activity is thermodynamically inhibited. When the electrical field is dissipated by an ionophore-mediated transport of ions out of the proteoliposomes along the electrical gradient, thermodynamic backup pressure is released and enzymatic activity is stimulated. This is known as respiratory control and was indeed observed. Ech2 activity was slightly stimulated by 16% (TCS) or 11% (ETH2120) with a H_2_ evolution rate of 0.64 ± 0.17 U/mg or 0.60 ± 0.07 compared with 0.53 ± 0.11 U/mg in the control, demonstrating respiratory control (Fig. 3*B*) and the buildup of an electrical field across the proteoliposomal membrane.

### Ech2 activity establishes a transmembrane electrochemical H^+^ gradient

Finally, to prove that Ech2 indeed is a chemiosmotic coupling site, we aimed to directly demonstrate ion transport dependent on Ech activity in proteoliposomes. To test for possible Na^+^ transport, proteoliposomes were preincubated with 1 μCi/ml (carrier-free) ^22^NaCl. Upon addition of pyruvate [reduction of Fd by pyruvate:ferredoxin oxidoreductase (PFOR)], there was very little uptake of ^22^Na^+^ that was indistinguishable from the assay performed in the absence of Fd (Fig. 3*C*). The same trend was observed when proteoliposomes were preincubated with TCS or ETH2120, respectively. Combined with data shown above, these experiments do not favor the idea of Na^+^ translocation coupled to Ech2 activity.

To test for a possible H^+^ transport, the generation of a ΔpH was studied using the fluorescence pH indicator ACMA. Therefore, reconstituted proteoliposomes were preincubated with ACMA and the reaction was started by addition of pyruvate, which immediately led to the reduction of Fd by PFOR. This led to a continuous decrease of ≈20% in fluorescence (quench), demonstrating ΔpH formation due to H^+^ transport into the vesicle lumen (Fig. 3*D*, black). After reaching a plateau, the protonophore TCS was added and the ΔpH was dissipated immediately (Fig. 3*D*). The dissipation of ΔpH was not observed when ETH2120 was added instead of TCS (data not shown). Addition of solvent (EtOH) had no effect on the ΔpH formed (Fig. 3*D*, green). The preincubation of ETH2120 in the presence of NaCl even stimulated H^+^ transport sightly, again demonstrating respiratory control (Fig. 3*D*, brown). In contrast, when proteoliposomes were preincubated with ETH2120 without any addition of NaCl no stimulation of ΔpH was observed (Fig. 3*D*, pink). If only half the amount of protein was added, the resulting ΔpH was less pronounced (≈10%) (Fig. 3*D*, yellow). Furthermore, the ΔpH formation was inhibited by DCCD (brown) and also not observed in liposomes without Ech2 (blue) or in the absence of Fd (gray) (Fig. 3*D*). Preincubation of the proteoliposomes with the protonophore TCS before the start of reaction did not lead to quenching or dequenching (Fig. 3*D*, red). In summary, the experiments clearly showed that Ech2 activity leads to the establishment of a ΔpH but not a ΔpNa^+^.

**Figure 4:**
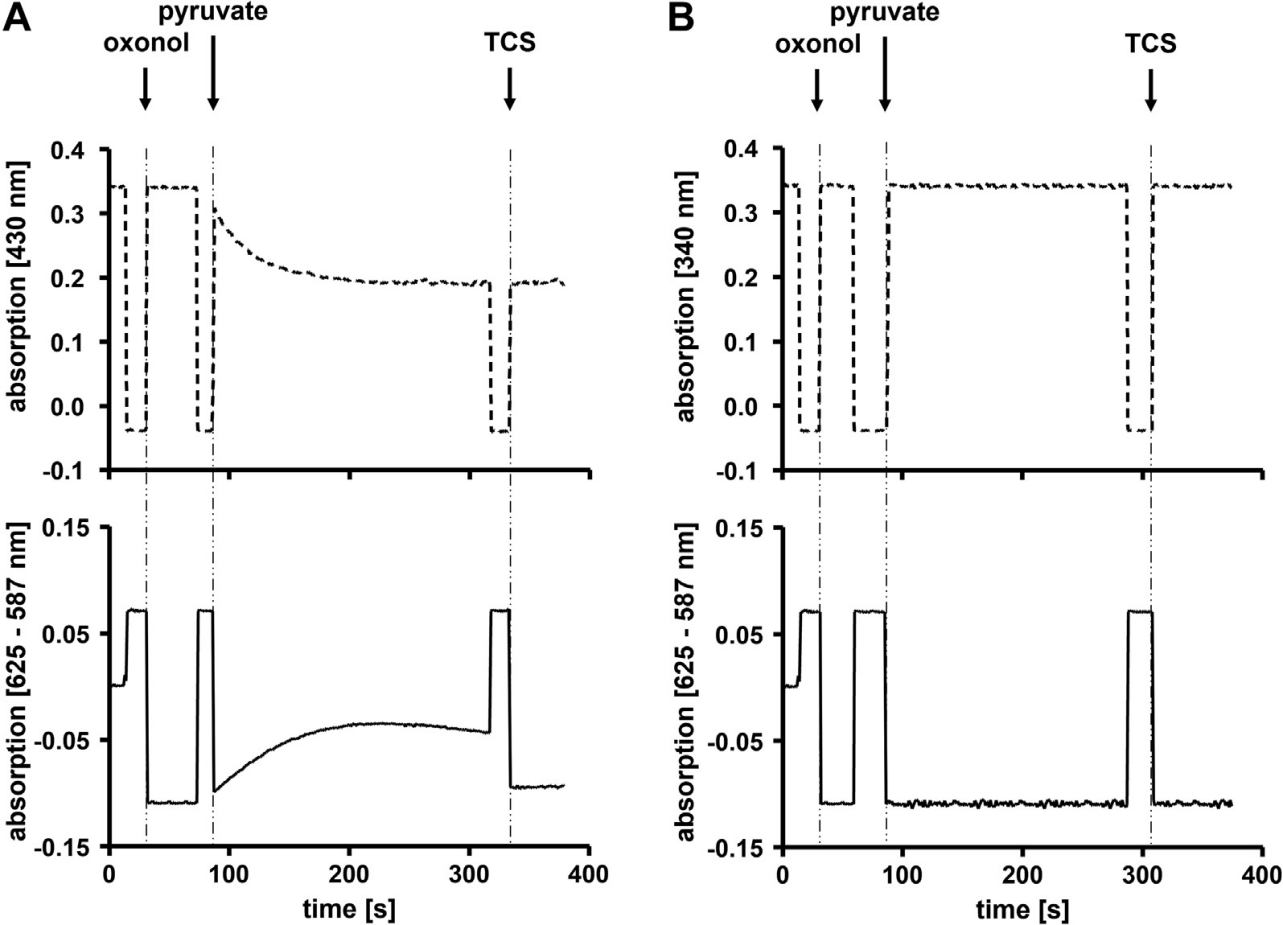
**Ech2-catalyzed H**^**+**^**transport is electrogenic.** The generation of a membrane potential was recorded by measuring the difference in absorption changes (625–587 nm) of oxonol VI and the reduction of Fd (430 nm) simultaneously. The measurements were performed as described in Experimental procedures. *A*, to induce the establishment of an electrical field, the assay containing 200 μg proteoliposomes was started with 10 mM pyruvate. To dissipate the electrical field 30 μM TCS was added as indicated. *B*, control assays omitted Fd. N = 3 independent experiments.

To demonstrate that H^+^ transport by Ech2 is electrogenic, we used the voltage-sensitive dye oxonol VI to determine a membrane potential (Δψ) across the proteoliposomal membrane. When the reaction was started by the addition of pyruvate (reduction of Fd by PFOR was simultaneously monitored at 430 nm), an increase in oxonol VI absorption at 625 to 587 nm was detected, indicating the buildup of an electrical field across the proteoliposomal membrane (Fig. 4*A*). The increase in oxonol VI absorption leveled off after about 100 s, suggesting that formation of the gradient was limited by the proton leak (Fig. 4*A*). Consistently, TCS induced a rapid collapse of the electrical field (Figs 4*A* and S6*B*). The electrical field could only be established when reduced Fd as electron donor was present. When Fd was omitted in the assay, no increase of oxonol VI absorption at 625 to 587 nm was observed (Fig. 4*B*). Addition of ETH2120 (without additional NaCl) (Fig. S6*D*) or the solvent ethanol had no effect on the Δψ formed (Fig. S6*A*). In contrast, the addition of DCCD completely blocked the generation of a membrane potential (Fig. S6*C*). The same was observed when proteoliposomes were preincubated with ETH2120 and NaCl (Fig. S6*E*). Combined, these experiments validate that proton transport by Ech2 is electrogenic.

## Discussion

[Ni-Fe] hydrogenases are widespread in aerobic and anaerobic bacteria (1, 3, 6). They are classified into four groups, the uptake [Ni-Fe] hydrogenases (group 1), cyanobacterial uptake [Ni-Fe] hydrogenases and H_2_ sensors (group 2), bidirectional heteromultimeric cytoplasmic [Ni-Fe] hydrogenases (group 3), and H_2_-evolving, membrane-associated and energy-converting hydrogenases (group 4) (1, 3, 6, 7). This group is further subclassified into at least nine different subgroups (1, 3, 6). They all have undoubtedly in common a membrane association, but energy conservation has not been demonstrated for any member of the subgroups. For example, subgroup 4a contains the formate hydrogenlyase, an enzyme well studied in *Escherichia coli* where its physiological function is to detoxify formate, produced by mixed acid fermentation, by oxidation to CO_2_ and H_2_ (53, 54, 55, 56). This reaction has been discussed for a long time to be energy conserving, *i.e.*, coupled to ion transport across the membrane thus establishing an ion gradient that in turn could drive ATP synthesis (54, 55, 56, 57). Although this assumption has made it nearly into textbook knowledge, data for this assumption have never been provided.

This is different in other subclasses. Pioneering work in the methanogenic archaea *Methanosarcina mazei* and *Methanosarcina barkeri* not only purified and characterized a Fd^2-^-dependent, H^+^-reducing enzyme complex but also revealed that this activity is present in inverted membrane versicles and coupled to H^+^ translocation across the vesicular membrane (4, 18, 19, 27, 28). Although this group (4e, six-subunit core enzyme complex) has been initially studied in methanogenic archaea, it is also widespread in bacteria (1, 3, 6, 7). Another subgroup is the Fd^2-^ oxidizing, Mrp-linked group of energy-converting hydrogenases as present, for example, in the hyperthermophilic archaea *Pyrococcus furiosus* (32, 58) or *Thermococcus onnurineus* (59, 60). Again, enzymes have been purified and even the structure of the 14-subunit Mbh complex of *P. furiosus* was solved (32), but ion transport has not been demonstrated with a purified enzyme. However, membrane vesicles of *T. onnurineus* were shown to catalyze H^+^ as well as Na^+^ transport coupled to the Fd^2-^-dependent reduction of H^+^, consistent with the presence of an Mrp domain and the presence of a Na^+^-dependent ATP synthase (59, 61). The same was found in *P. furiosus* (48, 62).

The thermophilic acetogen *T. kivui* harbors to different *ech* gene cluster and the encoded proteins are classified in subgroup 4C (Ech1, CO-oxidizing Ech) and 4G (Ech2, Fd^2-^-oxidizing Ech) (17). *T. kivui* can grow on CO and experiments with whole cells as well as inverted membrane vesicles revealed H^+^ and Na^+^ transport coupled to CO oxidation (17, 63). Whether only one or both Ech complexes are involved remains to be established by biochemical and genetic methods.

Here, we have purified the eight-subunit Ech2 complex from *T. kivui* using a procedure previously used for the purification of Mbh (58) and Mbs (31) from *P. furiosus*. A plasmid-encoded His-tagged version of Ech2C was apparently assembled into the Ech2 complex encoded from the genome and allowed a one-step purification of the entire complex. The enzyme was inhibited by DCCD, a valuable tool for further analysis, that could help to identify the ion-translocating residues (46, 47, 48, 49, 50, 51). The complex could be reconstituted into liposomes with full retention of activity. Most important, Ech2-containing proteoliposomes neither required Na^+^ for activity nor were they able to translocate ^22^Na^+^. In contrast, electrogenic H^+^ transport was clearly demonstrated providing unequivocal evidence that Ech2 is a H^+^-translocating, respiratory enzyme. This is the first demonstration of ion transport coupled to H^+^ reduction in any energy-converting hydrogenases.

**Figure 5:**
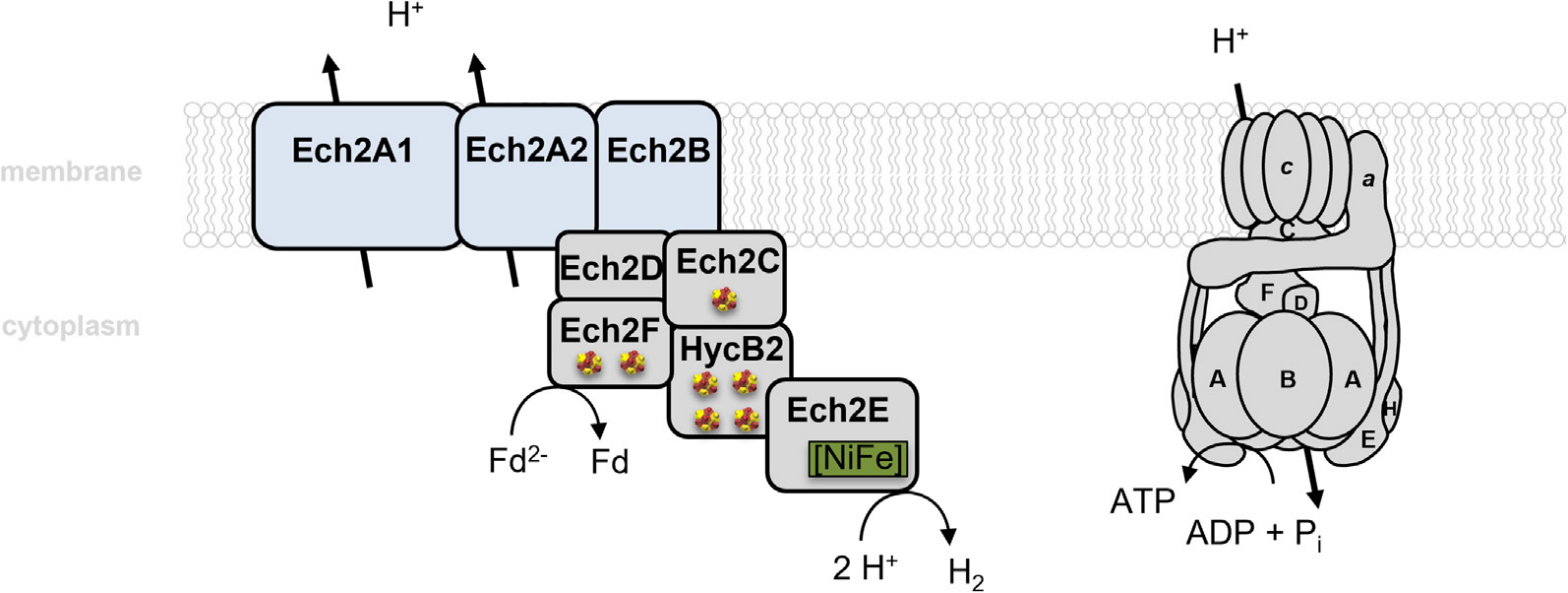
**The respiratory chain of the thermophilic acetogen *T. kivui*.** Schematic representation of the Ech2 complex and the ATP synthase of the respiratory chain in *T. kivui*. Exergonic electron transfer from reduced Fd to 2 H^+^ leads to the translocation of H^+^ across the cytoplasmic membrane and the electrochemical H^+^ potential is then the driving force for ATP synthesis. Membrane-bound subunits of Ech2 are colored blue. The electron pathway from the donor to the acceptor is unknown. Cubes, [4Fe-4S] clusters; [Ni-Fe], hydrogenase active site.

The potential difference between reduced Fd (E^0^′ [Fd^2−^/Fd] ∼−450 to −500 mV) and H^+^ (E^0^′ [H_2_/2H^+^] = −414 mV) is only E^0^′ = +86 mV to +36 mV and, thus, would only allow one H^+^ translocated per electron transported. For the ATP synthase of *T. kivui*, a H^+^/ATP stoichiometry of 3.6 is assumed (based on a number of 11 *c* subunits in the *c*-ring of the ATP synthase of *Clostridium paradoxum* (64, 65) and this would allow generation of 0.28 ATP per Fd^2-^ (Fig. 5). Despite low energy yields this respiratory system could enable microbial existence on early Earth at the thermodynamic limit of life.

## Experimental procedures

### Growth of *T. kivui*

*T. kivui* (DSM 2030) was grown at 66 °C in complex medium under anoxic conditions in 1-l or 20-l-bottles (Glasgerätebau Ochs) using 28 mM D-glucose as substrate (63). The medium was prepared using the anaerobic techniques as described previously (66, 67). Growth was monitored by measuring the absorbance at 600 nm. Plating and cultivation on solid media were the same as described previously (41). Kanamycin, 200 μg/ml, was used to select for recombinants.

### Cloning of *pMU131_ech2C-His*

Plasmid *pMU131_ech2C-His* was used for the expression of *ech2C* (TKV_c19700) in *T. kivui* (Fig. S7). The plasmid is based on plasmid *pMU131* (68), which replicates in *T. kivui* and confers resistance to kanamycin (10, 41). The insert *ech2C-His* (496 bp) was amplified using the primers Ech2C-His_for (3) and Ech2C-His_rev (4) (Table S1 and Fig. S7*C*). The backbone *pMU131* (7192 bp) was amplified using the primers pMU131_for (1) and pMU131_rev (2) (Table S1 and Fig. S7*B*), followed by the fusion of the PCR products *via* Gibson Assembly (Gibson Assembly Mastermix, NEB). *T. kivui* (DSM 2030) was transformed with the resulting plasmid *pMU131_ech2C-His* as described previously (41). Cells were plated on agar medium containing 28 mM glucose and 200 μg/ml kanamycin. To verify the transformation, colonies were picked and the transformed plasmids were checked using primer pairs seq1_for (5)/seq2_rev (6) (Table S1) binding on the *pMU131* backbone and amplifying the complete *ech2C-His* (Fig. S8).

## Purification of Ech2

All purification steps were performed under strictly anoxic conditions at room temperature in an anoxic chamber (Coy Laboratory Products) filled with 95 to 98% N_2_ and 2 to 5% H_2_. All buffers used were prepared using the anaerobic techniques as described previously (66, 67). *T. kivui pMU131_ech2C-His* cells were grown in 1- or 20-l-complex medium for 12 h to an *A*_600_ of around 2.2. Cells (2 g wet cell per 1-l-complex medium) were harvested and washed twice in buffer A (50 mM Tris/HCl, 150 mM NaCl, 20 mM MgSO_4_, 10 mM imidazole, 0.5 mM DTE, 4 μM resazurin, 5% [v/v] glycerol, pH 7.5). Afterward, the cells were resuspended in buffer A including 0.5 mM PMSF and 0.1 mg/ml DNAseI and passed once through a French pressure cell (110 Mpa). Cell debris was removed by centrifugation at 24,000*g* for 20 min at 4 °C. The crude extract was separated into the cytoplasmic and membrane fractions by ultracentrifugation at 208,057*g* for 1 h at 4 °C. The supernatant containing the cytoplasmic fraction was thoroughly decanted. The pelleted membranes were resuspended to homogeneity with a brush in buffer A to a final protein concentration of 10 mg/ml. Next, the detergent DDM was added at a final concentration of 1% [w/v] and the preparation was subsequently incubated under slight stirring overnight at 4 °C. The solubilized proteins were separated from the membrane fraction by ultracentrifugation at 208,057*g* for 45 min at 4 °C. The supernatant containing the solubilized proteins were added to a nickel nitrilotriacetic acid (Ni^2+^-NTA) resin (Qiagen) and incubated for 2 h at room temperature. Purification of the His-tagged Ech2 was carried out using a gravity flow column under anoxic conditions. The resin was washed with buffer B (50 mM Tris/HCl, 150 mM NaCl, 20 mM MgSO_4_, 30 mM imidazole, 0.5 mM DTE, 4 μM resazurin, 20% [v/v] glycerol, 0.02% [w/v] DDM, pH 7.5) to remove loosely bound proteins from the resin. Subsequently, specifically bound proteins were eluted by adding 150 mM imidazole-containing elution buffer C (50 mM Tris/HCl, 150 mM NaCl, 20 mM MgSO_4_, 150 mM imidazole, 0.5 mM DTE, 4 μM resazurin, 20% [v/v] glycerol, 0.02% [w/v] DDM, pH 7.5). Fractions containing Ech2 were collected, pooled, concentrated using 50-kDa VIVASPIN tubes and stored at 4 °C.

### Preparation of proteoliposomes

Proteoliposomes were prepared from L-α-phosphatidylcholine lipids (Merck). L-α-phosphatidylcholine, (1% [w/v]) was dissolved in buffer D (25 mM Hepes/KOH, 10 mM MgCl_2_, 2 mM DTE, pH 7.5), and proteoliposomes were generated by sonification using an ultrasonic homogenizer (Sonopuls GM mini20) and a sonotrode (Sonopuls MS73) with the following settings: total time, 20 min; amplitude, 30%; sonification time, 0.5 s; resting time, 0.5 s. Reconstitution of Ech2 into proteoliposomes was carried out using a modified protocol (62). The proteoliposomes were destabilized with 0.1% [w/v] DDM and purified Ech2 was added in a ratio of 1:5 [w/w]. The reconstitution mixture was incubated for 30 min at room temperature under gentle shaking. The detergent was removed stepwise using Bio-Beads (Bio-Rad Laboratories). First, Bio-Beads were added to a concentration of 30 mg/ml to the reconstitution mixture followed by an incubation at room temperature for 1 h. Afterward, the concentration of Bio-Beads was increased up to 60 mg/ml and the reconstitution mixture was incubated for an additional 1 h at room temperature. Last, the Bio-Beads concentration was again increased to 160 mg/ml and the reconstitution mixture was incubated for 12 h at 4 °C. Subsequently, the Bio-Beads were removed by filtering the mixture through a polypropylene column with a polyethylene filter (Machery-Nagel). The flow-through was centrifuged at 208,057*g* for 45 min at 4 °C to sediment the proteoliposomes. The proteoliposomes were washed once with buffer D, afterward resuspended in 0.5 ml of buffer D and stored at 4 °C. To verify impermeability of the proteoliposomes an artificial pH gradient with ammonium was established by resuspending 1:1 [v/v] of the proteoliposomes preparation in NH_4_Cl-containing buffer (10 mM Tris/HCl, 500 mM NH_4_Cl, 420 mM sucrose, 5 mM MgCl_2_, pH 8.0) overnight at 4 °C. The assay was performed in 1.4-ml quartz glass vials (Starna Typ 29-F, 4 × 10 mm light path, Starna GmbH). A volume of 10 μl of proteoliposomes was diluted in 1 ml choline buffer (10 mM Tris/HCl, 500 mM choline chloride, 420 mM sucrose, 5 mM MgCl_2_, pH 8.0) and the reaction was started by addition of 2.5 μM ACMA (solved in EtOH). The fluorescence of ACMA was measured in a fluorescence spectrophotometer (Hitachi F-4500 Fluorescence Spectrophotometer, Hitachi) with excitation at 410 nm and emission at 490 nm. The quench was abolished by 20 μl 1-butanol (100%).

### Measurement of Ech activity

All enzyme assays, unless otherwise specified, were performed in a N_2_ atmosphere (1 × 10^5^ Pa) at 66 °C in an overall liquid volume of 1 ml. One unit is defined as transfer of 2 μmol electrons/min. H_2_ evolution was assayed in 7.2-ml glass vials (Glasgerätebau Ochs GmbH) containing 100 to 200 μg membranes or 10 to 150 μg purified Ech2 in buffer E (50 mM Tris/HCl, 10 mM NaCl, 2 mM DTE, 4 μM resazurin, pH 8.0) or 50 to 950 μg proteoliposomes in buffer D. To reduce ferredoxin (Fd) (isolated from *Clostridium pasteurianum* (69)) the assay additionally contained 10 μg PFOR (isolated from *T. kivui* (10)), 400 μM coenzyme A (CoA), 30 μM Fd and 100 μM thiamine pyrophosphate (TPP). The reaction was started after a preincubation of the assay for 5 min at 66 °C by addition of pyruvate to a final concentration of 10 mM, if not otherwise specified. H_2_ was measured *via* gas chromatography as described previously (17). H_2_:Fd oxidoreductase activity was measured in 1.8-ml anoxic cuvettes (Glasgerätebau Ochs GmbH) sealed by rubber stoppers in a H_2_ atmosphere (2 × 10^5^ Pa). The assay contained 30 μM Fd in buffer F (50 mM CHES/NaOH, 10 mM NaCl, 2 mM DTE, 4 μM resazurin, pH 9.0). The reaction was started by addition of 15 to 20 μg purified Ech2. Fd was monitored spectrophotometrically at 430 nm (ε = 13.1 mM^-1^ cm^-1^). For K_m_ determination, the H_2_ and Fd concentrations ranged between 0 and 637 μM (in the aqueous phase) and 0 and 90 μM, respectively. For the determination of the pH and temperature profiles, the assay containing Ech2 was preincubated for 10 min at the pH or temperature indicated. The pH optima were determined in buffer G containing 50 mM MES, 50 mM CHES, 50 mM CAPS, 50 mM Bis-Tris, 50 mM Tris, 10 mM NaCl, 4 mM DTE, 4 μM resazurin at pH 5 to 10, as specified in the experiments. The effect of CO on the Fd^2-^:H^+^ oxidoreductase activity of Ech2 was measured with CO concentrations ranging between 0 and 964 μM (in the aqueous phase), respectively. The effect of ions (Na^+^, K^+^, Li^+^) on the Fd^2-^:H^+^ oxidoreductase activity of Ech2 was measured with desalted protein (final Na^+^ concentration in the assay: ≈180 μM). The buffer exchanged was performed using 50-kDa VIVASPIN tubes in anoxic conditions. After several concentration and dilution steps, Ech2 was stepwise transferred in buffer H (25 mM Tris/HCl, 2 mM DTE, pH 7.5). Afterward, the protein was preincubated 5 min at 66 °C in buffer H with 20 mM NaCl, 20 mM KCl or 20 mM LiCl, respectively, before starting the reaction. All measurements were performed in three biological triplicates.

### Inhibitor studies

For inhibition studies with DCCD 15 μg desalted Ech2 (final Na^+^ concentration in the assay: ≈180 μM) was preincubated in buffer H or buffer I (25 mM Bis-Tris/HCl, 2 mM DTE, pH 6) for 20 min at room temperature in presence or absence of 50 mM NaCl with 0 to 500 μM DCCD, respectively. Afterward, the Fd^2-^:H^+^ oxidoreductase activity assay was performed in buffer H as described above. Assays without the addition of NaCl were additionally supplemented with 50 mM NaCl just before starting the reaction. For inhibitor studies with the ionophores 3,3′,4′,5-tetrachlorosalicylanilide (TCS) and N,N,N′,N′-tetracyclohexyl-o-phenylendioxydiacetamid (ETH 2120) (30 μM each), 50 to 950 μg proteoliposomes were preincubated for 20 min at room temperature with 10 mM NaCl and the respective ionophore in buffer D, before Fd^2-^:H^+^ oxidoreductase activity assays were carried out as described above. All ionophores and inhibitors were dissolved in ethanol; controls received 1% [v/v] solvent only.

### Measurement of H^+^ translocation

Measurements of H^+^ translocation by Ech2 were performed under anoxic conditions in rubber stopper–sealed 1.4-ml quartz glass vials (Starna Typ 29-F, 4 × 10 mm light path, Starna GmbH) at a final liquid volume of 1 ml at 40 °C. Proteoliposomes, 240 or 120 μg and 2.5 μM ACMA (solved in EtOH) were preincubated for 10 min at room temperature in buffer D to ensure ACMA equilibrium. In control experiments proteoliposomes or liposomes were additionally preincubated with 1% [v/v] EtOH, 50 μM DCCD, 30 μM TCS, 30 μM ETH2120 and 10 mM NaCl, or 30 μM ETH2120 without the addition of NaCl, respectively. Afterward 10 μg PFOR (isolated from *T. kivui* (10)), 400 μM CoA, 30 μM Fd (isolated from *C. pasteurianum* (69)) and 100 μM TPP were added and the assay was incubated at 40 °C for 5 min. The reaction was started by addition of 10 mM pyruvate. The ACMA quench was abolished by addition of 30 μM TCS, if not otherwise specified. Fluorescence was measured in a fluorescence spectrophotometer (Hitachi F-4500 Fluorescence Spectrophotometer, Hitachi) with excitation at 410 nm and emission at 490 nm.

### Measurement of ^22^Na^+^ translocation

^22^Na^+^ translocation by Ech2 was analyzed under anoxic conditions in rubber stopper–sealed 3.5-ml glass vials at a final liquid volume of 1 ml at 40 °C. The assay contained 950 μg proteoliposomes, 10 μg PFOR (isolated from *T. kivui* (10)), 400 μM CoA, 30 μM Fd (isolated from *C. pasteurianum* (69)) and 100 μM TPP in buffer D. Carrier-free ^22^Na^+^ was added to a final concentration of 1 μCi/ml and incubated for 30 min at room temperature to ensure Na^+^ equilibrium before the reaction was started. Afterward, the assay was incubated at 40 °C for 5 min and the reaction was started by addition of 10 mM pyruvate. In control experiments Fd was omitted from the assay or proteoliposomes were additionally preincubated with 30 μM TCS or 30 μM ETH2120, respectively. The samples were taken and ^22^Na^+^ was “trapped” inside the proteoliposomes because uptake was separated from “external” ^22^Na^+^ using an electron exchange column as described previously (61). Afterward, proteoliposomes were denaturated and the “released” ^22^Na^+^ was detected by scintillation counting as described previously (61).

### Measurement of the membrane potential Δψ

The generation of a membrane potential (Δψ) was recorded by measuring the absorption changes (625–587 nm) of the potential-sensitive dye 1,5-bis(5-oxo-3-propylisoxazol-4-yl) pentamethine oxonol (oxonol VI). The measurements were performed in 1.8-ml anoxic cuvettes (Glasgerätebau Ochs GmbH) sealed with rubber stoppers in a final volume of 1 ml at 40 °C. The assay contained 200 μg proteoliposomes, 10 μg PFOR (isolated from *T. kivui* (10)), 400 μM CoA, 30 μM Fd (isolated from *C. pasteurianum* (8)), 100 μM TPP and 8 μM oxonol VI (solved in EtOH) in buffer D. The reaction was started by addition of pyruvate to a final concentration of 10 mM. To dissipate the electrical field, 30 μM TCS was added. In control experiments proteoliposomes were additionally preincubated with 1% [v/v] EtOH, 50 μM DCCD, 30 μM TCS, 30 μM ETH2120 and 10 mM NaCl, or 30 μM ETH2120 without the addition of NaCl, respectively.

### Analytical methods

The concentration of proteins was measured as described previously (70). Proteins were separated electrophoretically using denaturing or nondenaturing polyacrylamide gel electrophoresis (PAGE) according to Laemmli (1970) (71) or Wittig *et al.* (2007) (72) and stained with Coomassie brilliant blue G250. In-gel activity assay was performed as described previously (13). The iron content of the purified enzymes was determined by colorimetric methods (73). The Na^+^ concentration was determined using an Orion Star A214 sodium electrode (Thermo Scientific). The molecular mass of the purified Ech2 was determined using nondenaturing PAGE and defined size standards (thyroglobulin, 669 kDa; ferritin, 440 kDa; catalase, 250 kDa; lactate dehydrogenase, 140 kDa; albumin, 66 kDa; Amersham High Molecular Weight Calibration Kit, GE Healthcare). Peptide mass fingerprinting by MALDI-TOF was performed by the “Functional Genomics Center Zürich” at the ETH Zurich, Switzerland and results were analyzed using the Scaffold-Proteome Software version 4.10.0 (Proteome Software Inc).

## Data availability

All data are contained within the article.

## Supporting information

This article contains supporting information (10, 17, 69).

## Conflict of interest

The authors declare that they have no conflicts of interest with the contents of this article.
