# Supplementary material for: A purified energy-converting hydrogenase from Thermoanaerobacter kivui demonstrates coupled H+-translocation and reduction in vitro
Source: J Biol Chem. 2022 Jun 30;298(8):102216. doi: 10.1016/j.jbc.2022.102216 (PMC9356269; doi:10.1016/j.jbc.2022.102216)
Supplement: Supporting information [file mmc1.docx]

**Supporting information for:**

**A purified energy-converting hydrogenase from *Thermoanaerobacter kivui* demonstrates coupled H^+^-translocation and reduction *in vitro***

Alexander Katsyv^1^ and Volker Müller^1*^

^1^Department of Molecular Microbiology & Bioenergetics, Institute of Molecular Biosciences, Johann Wolfgang Goethe University, Frankfurt am Main, Germany

^*^Correspondence address: Prof. Volker Müller, Department of Molecular Microbiology & Bioenergetics, Institute of Molecular Biosciences, Johann Wolfgang Goethe University, Frankfurt am Main, Germany; Phone: 49-6979829507; Fax: 49-69-79829306;

E-mail: [vmueller@bio.uni-frankfurt.de](mailto:vmueller@bio.uni-frankfurt.de)

**This PDF file includes:**

Supplementary Figure S1 to S8

Supplementary Table S1

Supplementary References

**Supplementary Figures and Tables**


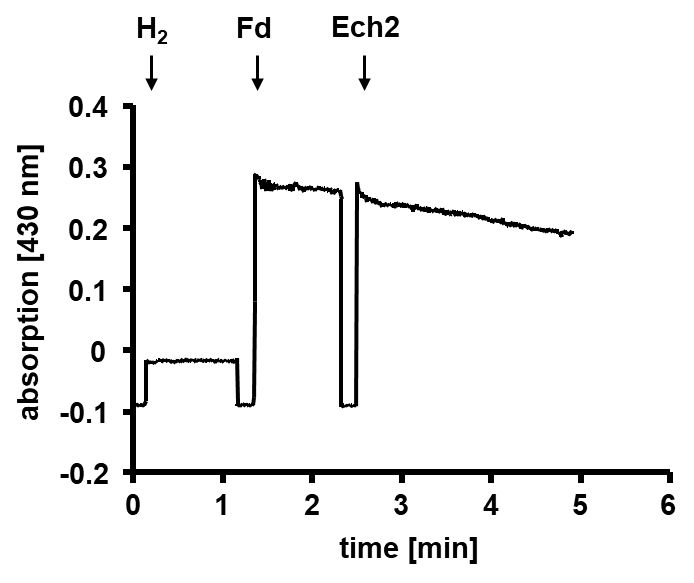


**Figure S1. H_2_-dependent Fd reduction catalyzed by Ech2.** H_2_:Fd oxidoreductase activity was measured in 1.8-ml anoxic cuvettes containing an overall liquid volume of 1 ml at 66 °C. The assay contained buffer F (50 mM CHES/NaOH, 10 mM NaCl, 2 mM DTE, 4 µM resazurin, pH 9.0), a 100% H_2_ atmosphere (2×10^5^ Pa) and 30 µM Fd (isolated from *C. pasteurianum* ([1](#_ENREF_8))). The reaction was started by addition of 15 μg Ech2. Reduction of Fd was monitored spectrophotometrically at 430 nm (ε = 13.1 mM^-1^ cm^-1^).


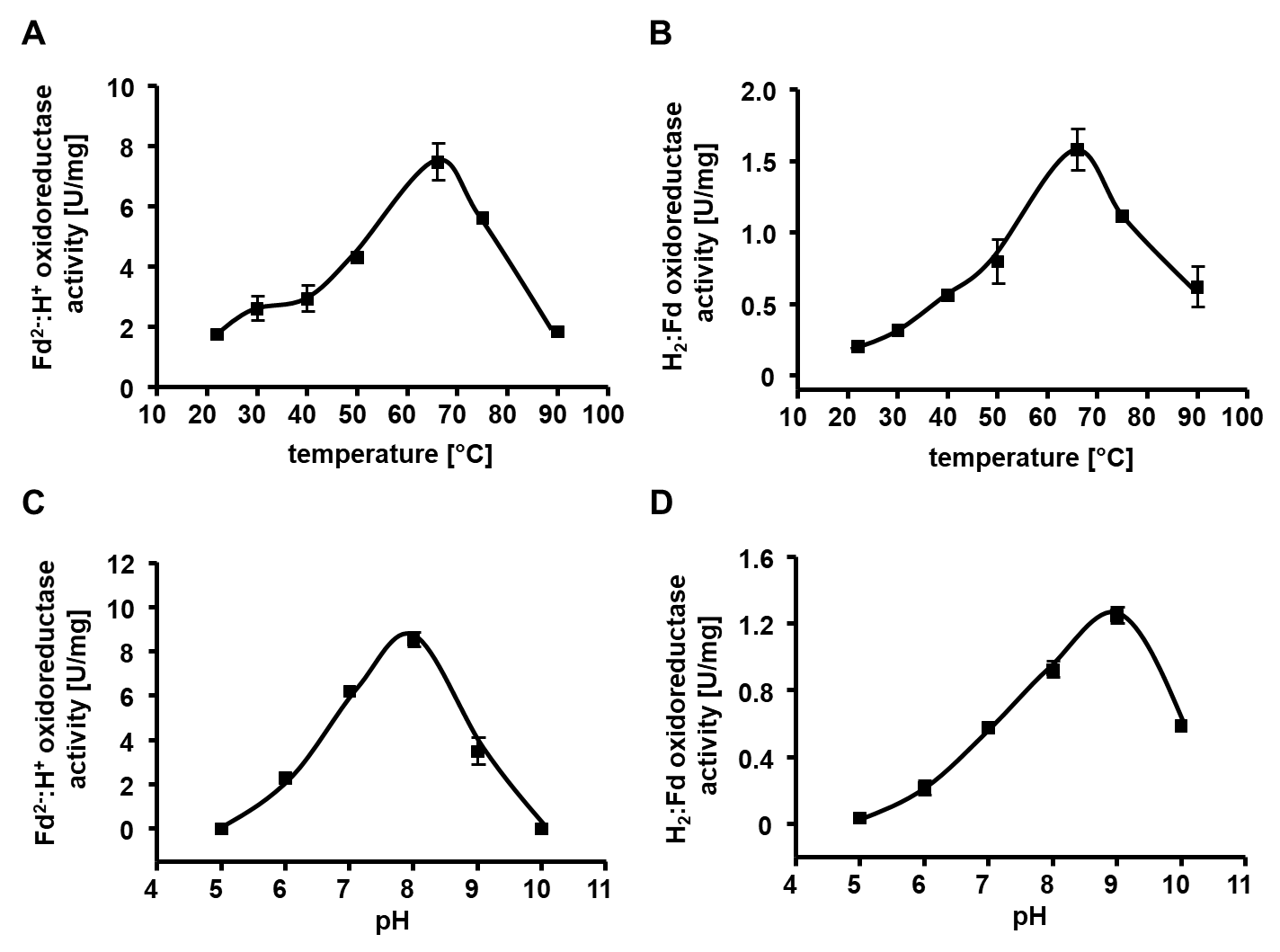


**Figure S2. pH optimum and temperature profile of purified Ech2.** Temperature **(A, B)** or pH **(C, D)** effect on the Ech2 activity was measured in 1.8-ml anoxic cuvettes or 7.2-ml glass vials containing an overall liquid volume of 1 ml at 22 - 85 °C **(A, B)** or 66 °C **(C, D)**, respectively. The Fd^2-^:H^+^ oxidoreductase activity assay contained buffer E (50 mM Tris/HCl, 10 mM NaCl, 2 mM DTE, 4 µM resazurin, pH 8.0) **(A)** or buffer G (50 mM MES, 50 mM CHES, 50 mM CAPS, 50 mM Bis-Tris, 50 mM Tris, 10 mM NaCl, 4 mM DTE, 4 μM resazurin, pH 5 - 10) **(C)**, 15 μg Ech2, 10 μg PFOR (isolated from *T. kivui* ([2](#_ENREF_6))), 400 μM CoA, 30 µM Fd (isolated from *C. pasteurianum* ([1](#_ENREF_8))), a 100% N_2_ atmosphere (1×10^5^ Pa) and 100 μM TPP. The reaction was started by addition of 10 mM pyruvate. H_2_ was measured *via* gas chromatography as described previously ([3](#_ENREF_9)). The H_2_:Fd oxidoreductase activity assay contained buffer F (50 mM CHES/NaOH, 10 mM NaCl, 2 mM DTE, 4 µM resazurin, pH 9.0) **(B)** or buffer G (50 mM MES, 50 mM CHES, 50 mM CAPS, 50 mM Bis-Tris, 50 mM Tris, 10 mM NaCl, 4 mM DTE, 4 μM resazurin, pH 5 - 10) **(D)**, 30 µM Fd and a 100% H_2_ atmosphere (2×10^5^ Pa). The reaction was started by addition of 15 μg Ech2. Reduction of Fd was monitored spectrophotometrically at 430 nm (ε = 13.1 mM^-1^ cm^-1^). The average of two measurements from one representative experiment out of two independent replicates is shown. Error bars represent the SEM.


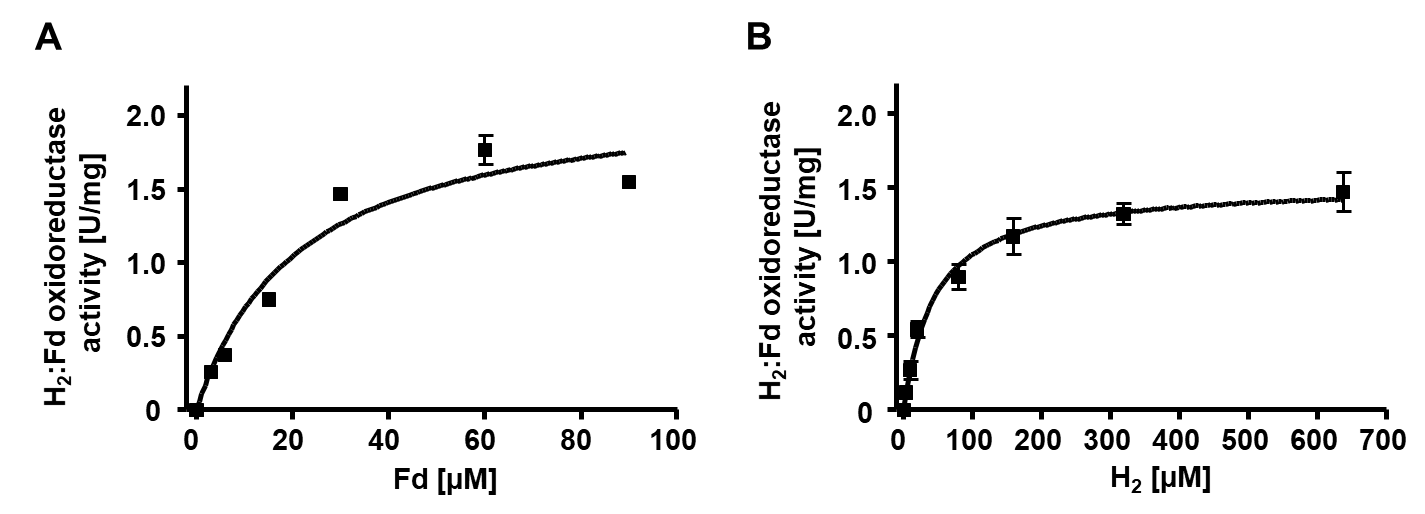


**Figure S3. Fd and H_2_ dependence of Ech2 activity.** H_2_:Fd oxidoreductase or Fd^2-^:H^+^ oxidoreductase activity was measured in 1.8-ml anoxic cuvettes or 7.2-ml glass vials containing an overall liquid volume of 1 ml at 66 °C. The H_2_:Fd oxidoreductase assay contained buffer F (50 mM CHES/NaOH, 10 mM NaCl, 2 mM DTE, 4 µM resazurin, pH 9.0) and different amounts of Fd (isolated from *C. pasteurianum* ([1](#_ENREF_8))) **(A)** or H_2_ in the aqueous phase **(B)**, respectively. The reaction was started by addition of 15 μg Ech2. Reduction of Fd was monitored spectrophotometrically at 430 nm (ε = 13.1 mM^-1^ cm^-1^). The average of two measurements from one representative experiment out of two independent replicates is shown. Error bars represent the SEM.


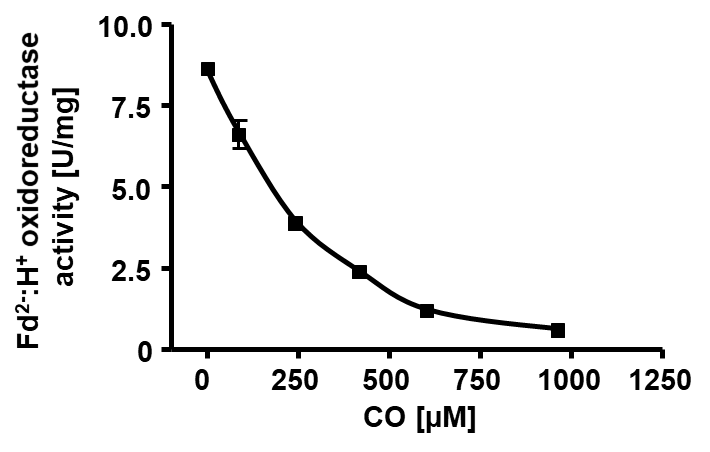


**Figure S4. CO inhibition of Ech2 activity.** Fd^2-^:H^+^ oxidoreductase activity was measured in 7.2-ml glass vials containing an overall liquid volume of 1 ml at 66 °C. The assay contained buffer E (50 mM Tris/HCl, 10 mM NaCl, 2 mM DTE, 4 µM resazurin, pH 8.0), 15 μg Ech2, 10 μg PFOR (isolated from *T. kivui* ([2](#_ENREF_6))), 400 μM CoA, 30 µM Fd (isolated from *C. pasteurianum* ([1](#_ENREF_8))), a 100% N_2_ atmosphere (1×10^5^ Pa), 100 μM TPP and different concentrations of CO in the aqueous phase, respectively. The reaction was started by addition of 10 mM pyruvate. H_2_ was measured *via* gas chromatography as described previously ([3](#_ENREF_9)). The average of two measurements from one representative experiment out of two independent replicates is shown. Error bars represent the SEM.


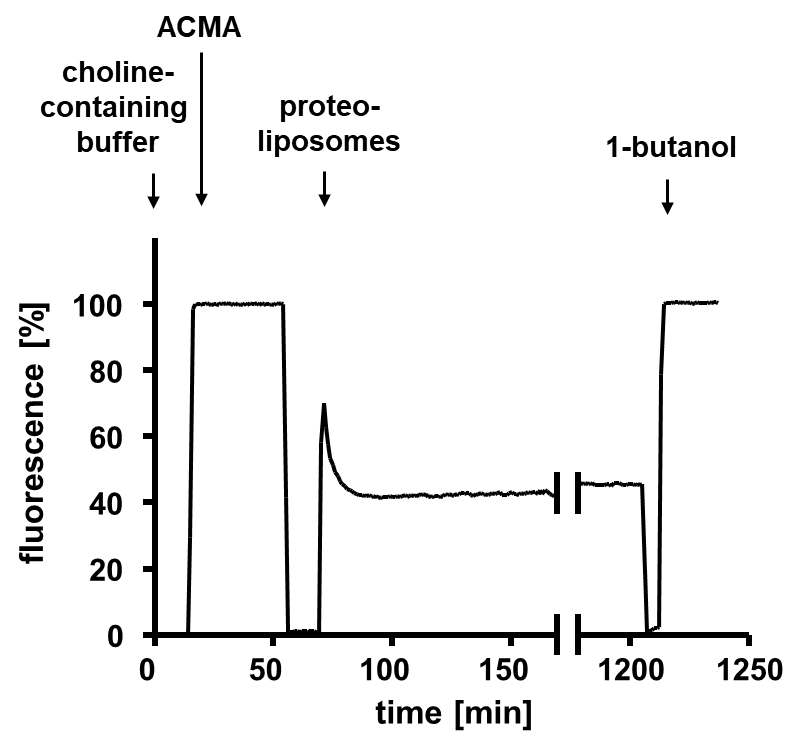


**Figure S5. Establishment of an artificial pH gradient in proteoliposomes.** To verify the impermeability of the reconstituted proteoliposomes an artificial pH gradient was established by resuspending 1:1 [v/v] of the proteoliposomes preparation in NH_4_Cl-containing buffer (10 mM Tris/HCl, 500 mM NH_4_Cl, 420 mM sucrose, 5 mM MgCl_2_, pH 8.0) over night at 4 °C. The assay was performed in 1.4-ml quartz glass vials. 10 µl of proteoliposomes were diluted in 1 ml choline-buffer (10 mM Tris/HCl, 500 mM choline chloride, 420 mM sucrose, 5 mM MgCl_2_, pH 8.0) and the assay was started by addition of 2.5 µM ACMA (solved in EtOH). The fluorescence of ACMA was measured in a fluorescence spectrophotometer with excitation at 410 nm and emission at 490 nm. The quench was abolished by 20 μl 1-butanol (100%).


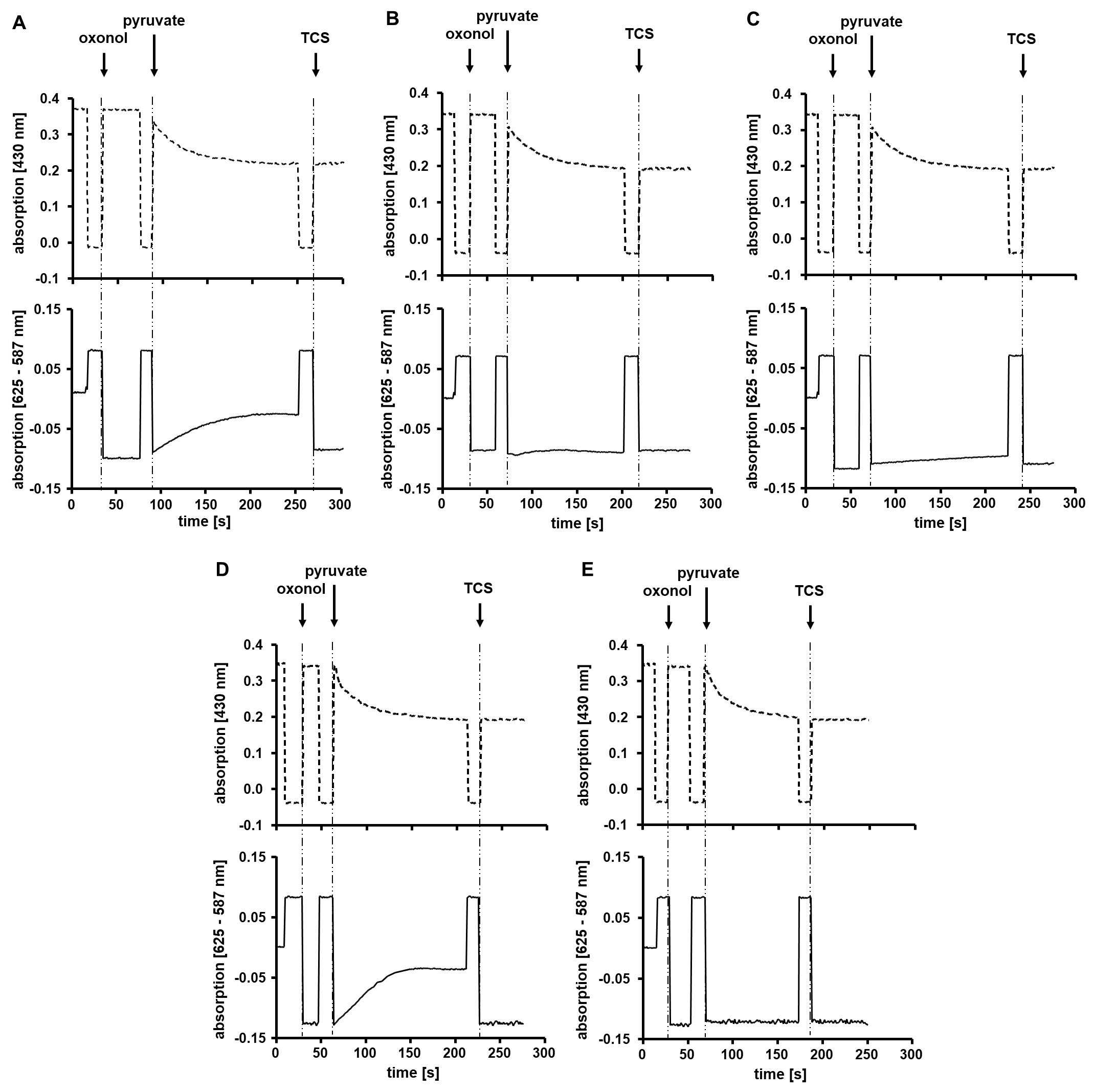
**Figure S6. Generation of Δψ is inhibited by addition of TCS and DCCD.** The measurements were performed in 1.8-ml anoxic cuvettes sealed with rubber stoppers in a final volume of 1 ml at 40 °C. The assays contained 200 µg proteoliposomes, 10 μg PFOR (isolated from *T. kivui* ([2](#_ENREF_6))), 400 μM CoA, 30 μM Fd (isolated from *C. pasteurianum* ([1](#_ENREF_8))), 100 μM TPP and 8 μM oxonol VI (solved in EtOH) in buffer D (25 mM HEPES, 10 mM MgCl_2_, 2 mM DTE, pH 7.5). Proteoliposomes were additionally pre-incubated with 1% EtOH **(A)**, 30 μM TCS **(B)**, 50 μM DCCD **(C),** 30 μM ETH2120 without the addition of NaCl **(D)** or 30 μM ETH2120 and 10 mM NaCl **(E)**, respectively. To induce the generation of an electrical field, the assay was supplemented with 10 mM pyruvate. To dissipate the field, 30 μM TCS was added as indicated. Absorbance of oxonol VI or Fd was measured as difference of 625 and 587 nm or 430 nm, simultaneously.

**
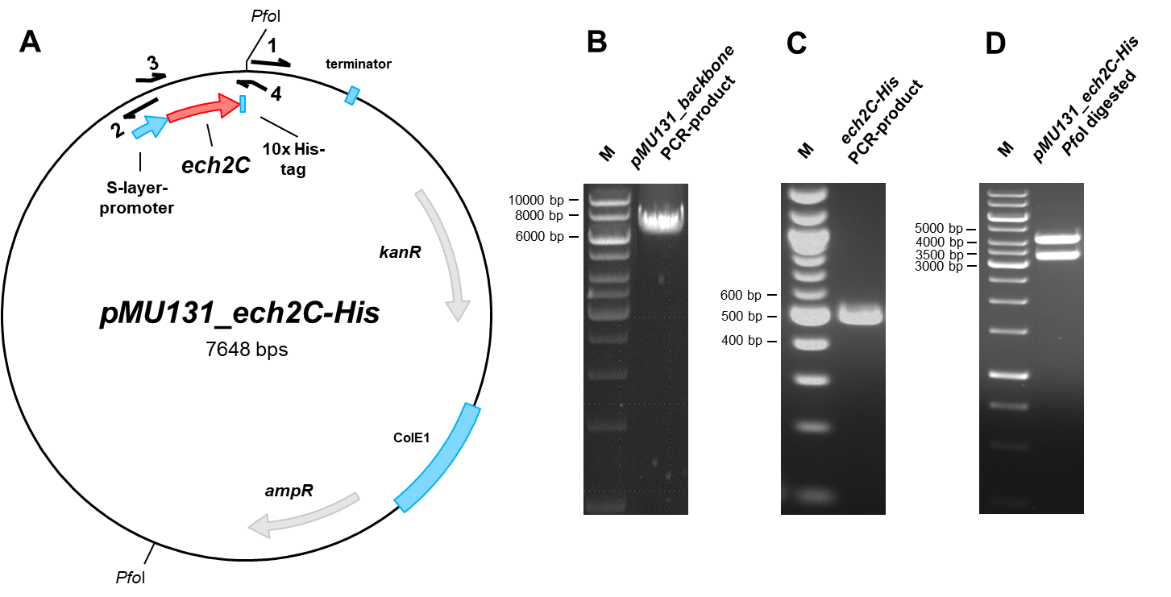
**

**Figure S7. Cloning of *pMU131_ech2C-His*.** **(A)** To purify a tagged Ech2 version in *T. kivui*, the construct *pMU131_ech2C-His* was cloned. **(B)** Therefore, *pMU131* backbone, including a S-layer-promoter, was amplified using corresponding primers (1, 2 (SI Appendix, Tab. S1)) *via* PCR. **(C)** *Ech2C-His* was amplified from genomic DNA of *T. kivui* *via* PCR, using corresponding primers (3, 4 (SI Appendix, Tab. S1)), containing an additional DNA sequence coding for a 10x His-tag. Amplified *ech2C-His* and *pMU131* were fused *via* Gibson Assembly and transformed in *E. coli* HB101. **(D)** Afterwards, plasmids were isolated and digested with *Pfo*I. The resulting sizes for *pMU131_ech2C-His* was 4241 bp and 3407 bp. M, Gene Ruler 1 kb DNA ladder.


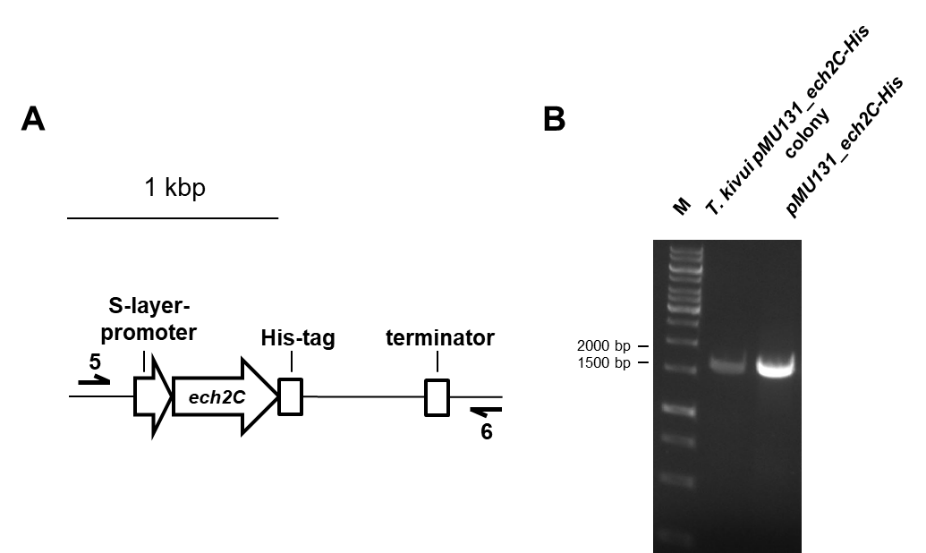


**Figure S8. Verification of the *pMU131_ech2C-His* construct.** To verify the nature of the plasmid *pMU131_ech2C-His* after propagation, *T. kivui* colonies were picked and the plasmids were checked by using primer pairs seq1_for (5)/ seq2_rev (6) (SI Appendix, Tab. S1) binding on the *pMU131* backbone and amplifying the complete *ech2C-His* sequence **(A)**. The resulting size was 1652 bp **(B)**. M, Gene Ruler 1 kb DNA ladder.

**Table S1. Primers used in this work.**

| **No.** | **Primer** | **Sequence (5’→ 3’)** |
| --- | --- | --- |
| 1 | pMU131_for | TTTTTTAAATTTATCCAGGATAAAAGAGAAGACTC |
| 2 | pMU131_rev | ACAGTCAATCCTCCTCCTTG |
| 3 | Ech2C-His_for | caaggaggaggattgactgtATGCTTGAACATTTTCGAG |
| 4 | Ech2C-His_rev | tcctggataaatttaaaaaaTCAATGATGATGATGATGGTGATGATGATGGTGTTCCTTTCTCAACTCCTTGAAAATC |
| 5 | seq1_for | TCTAACACAATTATATCATAAGGATTGATA |
| 6 | seq2_rev | AGTATTGTCAATATATTCAAGGCAA |

**SI References**

1. P. Schönheit, C. Wäscher, R. K. Thauer (1978) A rapid procedure for the purification of ferredoxin from *Clostridia* using polyethylenimine. *FEBS Lett.* **89**, 219-222.

2. A. Katsyv, M. C. Schoelmerich, M. Basen, V. Müller (2021) The pyruvate:ferredoxin oxidoreductase of the thermophilic acetogen, *Thermoanaerobacter kivui*. *FEBS Open Bio* **5**, 1332-1342.

3. M. C. Schoelmerich, V. Müller (2019) Energy conservation by a hydrogenase-dependent chemiosmotic mechanism in an ancient metabolic pathway. *Proc. Natl. Acad. Sci. U.S.A.* **116**, 6329-6334.
